# Surgical outcomes of totally extraperitoneal repair for inguinal hernia: A retrospective multicenter propensity score‐matched study

**DOI:** 10.1002/ags3.12443

**Published:** 2021-03-10

**Authors:** Yu Takeuchi, Tsuyoshi Etoh, Kosuke Suzuki, Tetsuji Ohyama, Takahiro Hiratsuka, Tetsuya Ishio, Mutsuhiro Kugimiya, Toshifumi Matsumoto, Seiichiro Kai, Toshio Bandoh, Kohei Shibata, Kentaro Iwaki, Kouichirou Tahara, Yuji Shigemitsu, Masafumi Inomata, the OITA Trial Group

**Affiliations:** ^1^ Department of Surgery JCHO Nankai Medical Center Oita Japan; ^2^ Department of Gastroenterological and Pediatric Surgery Oita University Faculty of Medicine Oita Japan; ^3^ Biostatistics Center Kurume University Fukuoka Japan; ^4^ Department of Surgery Arita Gastrointestinal Hospital Oita Japan; ^5^ Department of Surgery Kitsuki City Yamaga Hospital Oita Japan; ^6^ Department of Surgery Almeida Memorial Hospital Oita Japan; ^7^ Department of Surgery National Hospital Organization Beppu Medical Center Oita Japan; ^8^ Department of Surgery Nakatsu Municipal Hospital Oita Japan; ^9^ Department of Surgery Oita Prefectural Hospital Oita Japan; ^10^ Department of Gastrointestinal Surgery Oitaken Kouseiren Tsurumi Hospital Oita Japan; ^11^ Department of Surgery Oita Red Cross Hospital Oita Japan; ^12^ Department of Surgery Oita Medical Center Oita Japan; ^13^ Department of Surgery Zeze Hospital Oita Japan

**Keywords:** inguinal hernia, open mesh repair, recurrence rate, TEP

## Abstract

**Background:**

Laparoscopic surgical approaches, including total extraperitoneal repair (TEP), have been widely accepted for inguinal hernia repair in Japan. However, there are limited data regarding recurrence after TEP in Japan, given the limited versatility of this procedure. This study retrospectively evaluated the rates of hernia recurrence after TEP and open mesh repair at multiple Japanese centers.

**Methods:**

This retrospective study evaluated 1917 patients who underwent inguinal hernia repair at 32 institutions in the Oita prefecture between January 2014 and December 2015. Eligible patients were grouped according to whether they underwent TEP (1011 patients) or open mesh repair (636 patients). Propensity score matching was performed 1:1 (total: 1076 patients, 538 patients from each group). The outcomes of interest were recurrence, morbidity, and postoperative recovery.

**Results:**

The TEP and open mesh repair groups had similar baseline characteristics. After propensity score matching, there was no significant difference between the two groups in terms of recurrence rate (TEP: 0.5% vs open mesh repair: 1.0%, P = .375). However, the TEP group had significantly longer operating times (median: 70.2 min vs 65.0 min, P < .001), significantly less blood loss (0‐5.1 mL vs 0–20.4 mL, P < .001), and significantly shorter postoperative hospital stays (median: 5.0 days vs 6.4 days, P < .001). The overall incidences of morbidity were 6.2% in the TEP group and 7.2% in the open mesh repair group (P = .535).

**Conclusion:**

This multicenter retrospective study with propensity score matching revealed that the recurrence rates were similarly low for TEP and open mesh repair of inguinal hernia. Thus, a well‐trained surgical team could use TEP as a standard procedure.

## Graphical abstract

The present study was performed to evaluate the recurrence rates and the overall incidences of morbidity after TEP and open mesh repair of inguinal hernia, using retrospective data from multiple institutions with propensity score matching in our region (Oita prefecture, Japan). TEP provides a low recurrence rate and a low incidence of overall morbidity.

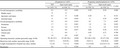

## 1 INTRODUCTION

Inguinal hernia repair is one of the most common procedures performed by general surgeons throughout the world.^1^ Most patients who experience inguinal swelling or discomfort visit a hospital and undergo surgery at the same institution, which can even be performed at small clinics in Japan. Conventional tissue‐based repair was historically the standard option for inguinal hernia repair, although the use of prosthetic mesh has increased since Lichtenstein et al described a tension‐free repair using a Prolene mesh.^2^ In the 1990s, laparoscopic repair techniques were introduced for inguinal hernia repair, which included transabdominal preperitoneal repair (TAPP) and total extraperitoneal repair (TEP), and these techniques have become standardized and have grown in popularity.^3^, ^4^ Thus, the current inguinal hernia repair options can be grouped as conventional tissue‐based repair, open mesh repair, TAPP, and TEP,^5^ which are selected based on the surgeon's or institution's preference. The 13th Nationwide Survey of Endoscopic Surgery in Japan (2014–2015) revealed that laparoscopic surgery is preferred for inguinal hernia repair in 38.6% of hospitals, although TEP is only used in 18.8% of laparoscopic repairs.^6^

Recurrence after hernia repair surgery is also an important issue. However, there are limited data regarding hernia recurrence after TEP in Japan, given the limited versatility of this procedure. Therefore, this retrospective study aimed to evaluate the recurrence rates after TEP and open mesh repair of inguinal hernia, using data from multiple institutions in our region (Oita prefecture, Japan).

## 2 MATERIALS AND METHODS

### 2.1 Patients and data collection

This retrospective multicenter study evaluated 1917 patients who underwent inguinal hernia repair at 32 institutions in Oita prefecture between January 2014 and December 2015. However, we excluded 226 cases that involved bilateral hernia, 15 cases that involved TAPP, 26 cases that involved conventional tissue‐based repair, and three cases that involved other repair techniques. Thus, the study included 1647 patients who underwent either TEP or open mesh repair for unilateral inguinal hernia.

The operative method was selected based on the preferred strategy at each hospital. Open mesh repair was defined as anterior repair with mesh for groin hernia, various posterior repairs (Kugel, Direct Kugel, Prolene Hernia System), the mesh plug repair, and the Lichtenstein repair, because there are no comparative data for each procedure's recurrence rate. We included recurrent cases in this analysis because previous studies had reported no differences in the re‐recurrence rates after TEP and open mesh repair.^7^ Both TEP and open mesh repair procedures were performed by experienced surgeons who were certified by the Japan Surgical Society. Patient data were extracted from their medical records and our database of outpatient visits during a 2‐year follow‐up period. Baseline demographic characteristics included sex, age, body mass index (BMI; kg/m^2^), comorbidities (constipation, pulmonary disorders, and prostatectomy, which were considered risk factors for inguinal hernia), and anticoagulant use. In addition, we collected data regarding the hernia location, type, and initial or recurrent status. Clinical outcomes were defined as the operating time, blood loss, intraoperative complications, postoperative complications, postoperative hospital stay, and recurrence after surgery. Diagnosis of recurrence was made by physical examination to confirm the swelling of the inguinal region and computed tomography to confirm the presence of a hernia sac in the myopectineal orifice. Median follow‐up time of all patients was 24 mon. All complications were graded according to the Clavien–Dindo classification.

### 2.2 Ethical considerations

The study protocol was approved by the Oita University Faculty of Medicine Institutional Review Board (approval number: 1204). The confidentiality of patient data was protected according to our center's policies. All institutions applied for and obtained study approval from their respective Institutional Review Boards. At all participating institutions, the attending surgeons obtained written informed consent from each patient.

### 2.3 Surgical procedures

#### 2.3.1 Laparoscopic TEP

The TEP procedure was performed under general anesthesia using 3 ports: 1 port for the laparoscope placed just below the umbilicus and 2 ports placed on the lower abdominal midline. The extraperitoneal space was laparoscopically created using a balloon or blunt dissection and then insufflated using carbon dioxide with an extra‐pneumoperitoneum pressure of 8–10 mm Hg. The decision regarding complete or incomplete reduction of the hernia sac was made at the surgeon's discretion. In cases with incomplete sac reduction, the sac was ligated and divided at the level of the internal ring. A polypropylene monofilament mesh (at least 10 × 15 cm) was spread to cover the entire myopectineal orifice and attached using tacks.

#### 2.3.2 Open mesh repair

All open procedures were performed under general, lumbar, or local anesthesia. The mesh was placed at the anterior wall of the inguinal canal, the preperitoneal space, or both locations. To reinforce the anterior wall, a flat mesh was placed against the inguinal floor and its circumference was sutured to the pubic tubercle, the conjoint tendon, and the inguinal ligament (the Lichtenstein repair).^2^ A mesh‐plug repair involved placing an umbrella‐shaped mesh into the inguinal ring, in addition to the Lichtenstein repair.^8^ The Kugel or Direct Kugel repair techniques involved preperitoneal placement of a polypropylene mesh with a memory recoil ring.^9^ The Prolene Hernia System was used to combine the posterior and anterior approaches to reinforcement at the inguinal canal.^10^

### 2.4 Endpoints

The primary endpoint for the present study was hernia recurrence. The secondary endpoints were blood loss, operating time, morbidity, and postoperative hospital stay.

### 2.5 Statistical analysis

Based on expected differences in the baseline characteristics of patients who underwent TEP or open mesh repair, we used propensity score matching (PSM) to reduce the influence of confounding factors. Because there were missing data regarding some baseline covariates, we used the “within approach,”^11^ which is recommended when conducting PSM with incomplete data. First, multiple imputation via a chained equation was used to create 100 multiply imputed datasets, and PSM was performed for each dataset. The covariates included in the logistic regression model were sex, age, BMI, smoking habit, comorbidities (constipation, pulmonary disorders, and prostatectomy), anticoagulant use, ascites, contralateral operation, hernia location, hernia type, and initial or recurrent status. The nearest‐neighbor method was used without replacement within a caliper, and the caliper was set to 0.2 of the standard deviation of the logit for the estimated PSM. Unmatched patients were excluded. The balance of covariates between the two procedures was then assessed based on the absolute standardized difference:

$$d=100\times \frac{\left|{\bar{x}}_{1}‐{\bar{x}}_{2}\right|}{\sqrt{\left(s_{1}^{2}+s_{2}^{2}\right)/2}},$$

where ${\bar{x}}_{1}\;\text{and}\;{\bar{x}}_{2}$ are the groups' means, and $s_{1}^{2}\;\text{and}\;s_{2}^{2}$ are the groups' variances. For each dataset, we calculated the Wilcoxon statistics and their variances for continuous data, as well as the risk differences and their variances for binary data. Finally, the values from the 100 datasets were pooled using Rubin's rules.^12^

In addition to the PSM analyses, we performed univariate analyses for complete cases. All statistical analyses were performed using R software (v. 3.6.2; R Foundation, Vienna, Austria). All *P*‐values were two‐sided and differences were considered statistically significant at *P* < .05.

## 3 RESULTS

### 3.1 Patient characteristics

**FIGURE 1:**
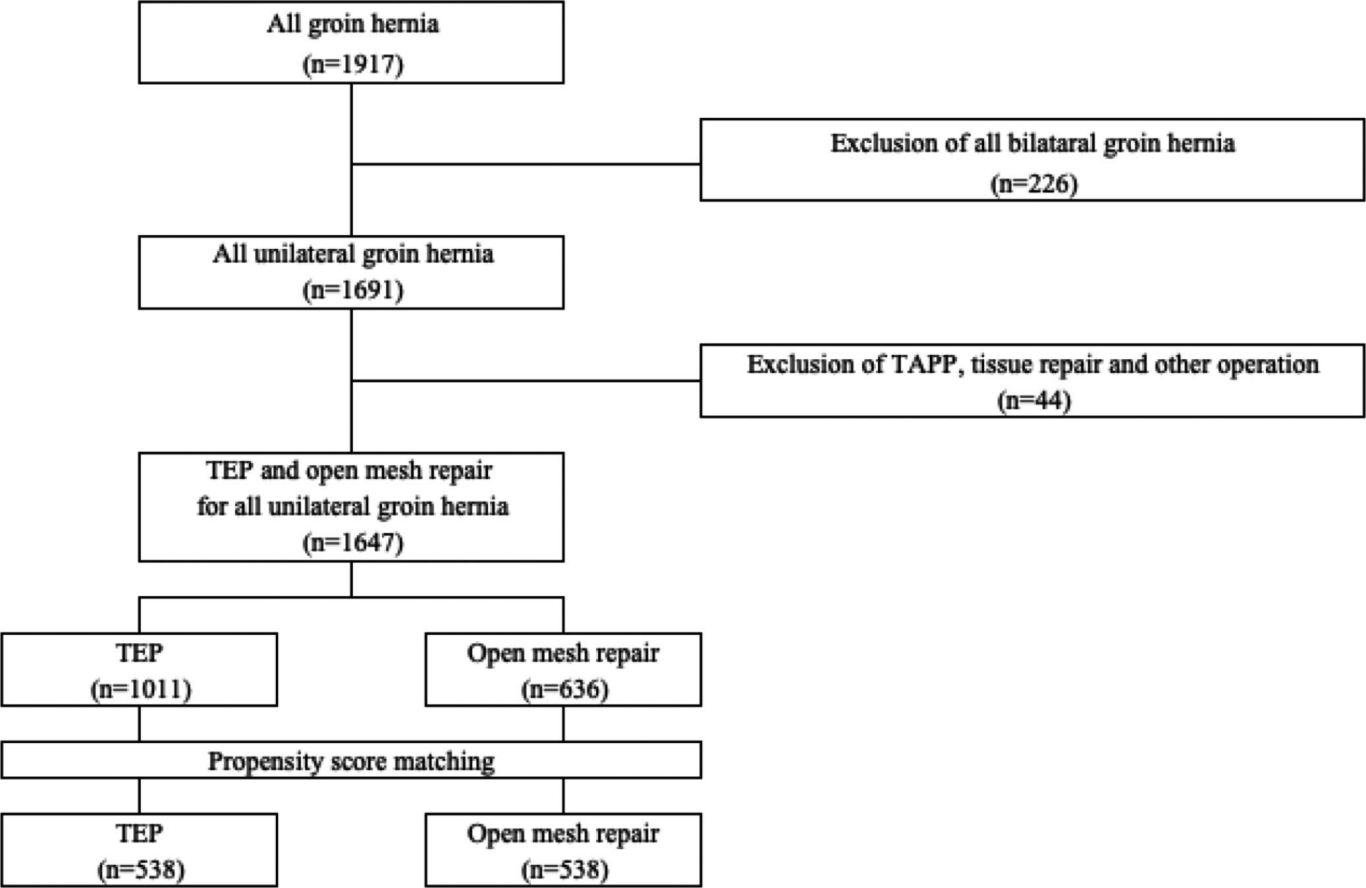
CONSORT flowchart

The CONSORT flowchart is shown in Figure 1 and the patients' baseline characteristics are shown in Table 1. In terms of the surgical procedure employed, TEP was performed in a median of 68.8% (median, range: 0%–95.0%) and open mesh repair in 30.4% (median, range: 2.0%–100%) of patients at each institution. Interestingly, 28.6% of institutions reported not performing TEP at all. The TEP group (1011 patients) and open mesh repair group (636 patients) had comparable baseline characteristics. After the PSM, 538 pairs of patients were analyzed and there were no significant differences in their characteristics.

**TABLE 1:** Baseline characteristics before and after propensity score matching

		All patients (n = 1647)					Propensity‐matched patients (n = 1076)		
		TEP (n = 1011)	%	Open mesh repair (n = 636)	%	Absolute standardized difference	TEP (n = 538, %)	Open mesh repair (n = 538, %)	Absolute standardized difference
Sex	Male	886	87.6	577	90.7				
	Female	125	12.4	59	9.3	9.94	11.0	10.8	1.83
Age	mean ± SD	66.9 ± 14.9		69.9 ± 12.5		21.9	68.8	69.5	5.46
BMI	mean ± SD	22.9 ± 2.9		22.8 ± 3.0		2.78	22.8	22.7	4.13
Life history	Smoking	314	31.1	182	28.6	3.53	32.8	34.4	3.11
Comorbidity	Constipation	106	10.5	70	11.0	1.68	10.8	10.6	2.40
	Pulmonary disorder	61	6.0	74	11.6	19.8	10.8	10.8	0.60
	Prostatectomy	14	1.4	87	13.7	47.9	2.6	2.8	1.15
	Anticoagulant	189	18.7	129	20.3	4.01	19.5	19.6	1.40
	Ascites	8	0.8	15	2.4	12.6	1.5	1.9	2.89
Contralateral operation		103	10.2	74	11.6	4.64	10.6	10.0	2.45
Location	Left	419	41.4	270	42.5				
	Right	592	58.6	366	57.5	2.04	58.6	57.4	2.63
Type	Lateral	775	76.7	480	75.5	2.48	77.3	75.9	2.57
	Medial	144	14.2	108	17.0	7.74	16.3	17.8	3.08
	Femoral	46	4.5	19	3.0	8.25	3.4	3.3	3.96
	Other	23	2.3	12	1.9	1.35	3.1	2.8	2.07
	Missing	20	2.0	14	2.2				
Initial/Recurrence	Initial	986	97.5	589	92.6				
	Recurrence	25	2.5	47	7.4	22.8	4.6	5.2	2.18

Notation of propensity‐matched patients is described in percent except age and BMI.

### 3.2 Recurrence rate

Recurrence was detected in six patients (0.6%) in the TEP group and seven patients (1.1%) in the open mesh repair group. After PSM, the recurrence rate was not significantly different between the two groups (0.5% vs 1.0%, *P* = .375) (Table 2).

**TABLE 2:** Surgical outcomes before and after propensity score matching

	All patients (n = 1647)			Propensity‐matched patients (n = 1076)		
	TEP	Open mesh repair	*P* value	TEP	Open mesh repair	*P* value
	% (n = 1011)	% (n = 636)		% (n = 538)	% (n = 538)	
Overall intraoperative morbidity	0.3	0.5	.682	0.28	0.34	.884
Bladder injury	0	0				
Spermatic cord injury	0.3	0	.288	0.28	0	.321
Intestinal injury	0	0.5	.054	0	0.34	.205
Overall postoperative morbidity	5.9	6.9	.474	6.2	7.3	.497
Hematoma	1.7	2.2	.461	1.8	2.6	.443
Seroma	1.4	2.4	.178	1.4	2.6	.222
Superficial SSI	0.1	0.6	.077	0.05	0.38	.325
Chronic pain	1	0.9	1	0.95	0.71	.708
Other	1.8	0.9	.207	1.9	1.1	.325
Operating time (min), median (percentile range 10–90)	70 (46–123)	67 (38–126)	<.001	70.2 (46.2–126)	65 (37‐124)	<.001
Blood loss (mL), median (percentile range 10–90)	0 (0–5)	0 (0–20)	<.001	0 (0–5.1)	0 (0‐20.4)	<.001
Length of postoperative hospital stay (days), median (percentile range 10–90)	5 (1–8)	7 (3–11)	<.001	5 (1.8–8.0)	6.4 (3.0‐11.5)	<.001
Recurrence	0.6	1.1	.266	0.5	1.0	.375

### 3.3 Morbidity and postoperative recovery

The overall incidences of morbidity were 6.2% in the TEP group and 7.2% in the open mesh repair group (*P* = .535). There were no significant differences in the incidences of hematoma (TEP: 1.8% vs open mesh repair: 2.6%, *P* = .443), seroma (TEP: 1.4% vs open mesh repair: 2.6%, *P* = .222), or chronic pain (TEP: 0.95% vs open mesh repair: 0.71%, *P* = .708). However, the TEP group had significantly longer operating times (median: 70.2 min vs 65.0 min, *P* < .001), significantly less blood loss (median: 0 mL [10–90th percentile: 0–5.1 mL] vs 0 mL [10–90th percentile: 0–20.4 mL] *P* < .001), and significantly shorter hospital stays (median: 5.0 days vs 6.4 days, *P* < .001) (Table 2).

## 4 DISCUSSION

Recurrence after hernia repair is an important issue, and this study revealed that TEP was associated with a low recurrence rate (0.6%). In contrast, a systematic review using updated traditional and cumulative meta‐analysis of randomized controlled trials revealed that recurrence was more common in the TEP group than in the Lichtenstein group (6% vs 4%).^13^ The 13th Nationwide Survey of Endoscopic Surgery in Japan (2014–2015) also indicated that the recurrence rate after TEP was 3.4%.^6^ However, we observed a lower recurrence rate for TEP relative to the reported results from randomized controlled trials^14^, ^15^ or the Japanese survey. We also observed a low incidence of complications after TEP. Therefore, TEP might be safe and effective in terms of reducing the risk of recurrence and improving the patient's postoperative recovery.

The 2015 update to the International Endohernia Society guidelines indicated that there was strong evidence that both TEP and TAPP are effective laparoscopic techniques for inguinal hernia repair.^16^ Furthermore, the HerniaSurge Group, which is an expert group of international surgeons that is working to establish international guidelines for groin hernia management, recommends that the choice of the technique should be based on the surgeon's skills, education, and experience. In this context, surgeon experience is considered a major risk factor for hernia recurrence after TEP, and improvements in surgical technique are important for preventing recurrence.^17^ In Japan, TEP is not a major laparoscopic procedure, as the 13th Nationwide Survey of Endoscopic Surgery in Japan revealed that TEP was performed for only 18% of laparoscopic repairs (laparoscopic repair was performed for approximately 45% of 59614 patients who underwent inguinal hernioplasties). However, in the Oita prefectural region (the setting for the present study), 20 of 36 institutions (56%) had TEP as their first choice for inguinal hernia repair. This may be related to TEP facilitating accurate diagnosis and anatomical visualization for the surgeon and assistant, which may help ensure that the myopectineal orifice is recognized and sufficiently covered with mesh.^18^ Another report has indicated that low‐volume surgeons (<25 vs ≥25 procedures/year) had a significantly higher recurrence rate after laparoendoscopic inguinal hernia repair,^19^, ^20^ which may suggest that the increased use of TEP helped reduce the recurrence rate in our region. Moreover, all procedures were performed by experienced surgeons who were certified by the Japan Surgical Society, which may also have contributed to the good outcomes that we observed.

Several randomized controlled trials have compared the recurrence rates of TEP, TAPP, and the Lichtenstein method, although the results have not supported a definitive conclusion (Table 3).^1^, ^26^ For example, an updated meta‐analysis revealed that the recurrence rate was significantly higher for TEP than for the Lichtenstein method.^13^ The present study revealed that open mesh repair had a low recurrence rate (1.1%), which agrees with previous reports. However, relative to the Lichtenstein method, the TEP group had significantly lower rates of postoperative hematoma formation, local paresthesia, and time to return of usual activities. In addition, TEP is associated with intraoperative complications, such as vascular injuries, and a longer operating time.^27^ Thus, the guidelines recommend the Lichtenstein technique as the standard procedure for open mesh repair.^1^ Nevertheless, in our region the Lichtenstein technique was not commonly used, and the mesh plug, Kugel, or Direct Kugel methods were more common. Similarly, the 13th Nationwide Survey of Endoscopic Surgery in Japan revealed that 88% of open mesh repairs involved the mesh plug, Kugel, or Direct Kugel methods. It is interesting that both TEP and open mesh repair provided acceptable results, which might be related to anatomical knowledge gained via TEP being applied during open mesh repair.

**TABLE 3:** Recurrence rate in TEP/TAPP and open mesh repair

Author	Year	Study design	n				Recurrence rate (%)		*P* value
			TEP/TAPP		Open (Procedure)		TEP/TAPP	Open	
Andersson B [21]	2003	RCT	81	TEP	87	L	2.6	0	.23
Douek M [23]	2003	RCT	122	TAPP	120		1.6	2.5	NS
Neumayer L [14]	2004	RCT	862	TEP; 90%, TAPP; 10%	834	L	10.1	4.9	NS
Hallen M [22]	2008	RCT	73	TEP	81	L	4.3	5.1	.37
Pokorny H [24]	2008	RCT	129	TEP; 36, TAPP; 93	69	L	TEP; 5.9, TAPP; 4.7	0	NS
Eklund AS [25]	2009	RCT	616	TEP	659	L	3.5	1.2	.008
Eker HH [17]	2012	RCT	235	TEP	222	L	4.9	8.1	.1
Miserez M [26]	2014	Meta‐analysis	1341	TEP, TAPP	1330	L	2.8	1.8	.12
The HerniaSurge Group [1]	2018	Meta‐analysis	1237	TEP, TAPP	1281	L	TEP;2.4, TAPP; 1.3	1.2	NS
Gutlic N [15]	2019	RCT	202	TEP	214	L	2.2	1	.36
Gavriilidis P [13]	2019	Meta‐analysis	2678	TEP	2790	L	6	4	.005
Present study		Retrospective (PSM)	538	TEP	538	L/MP/K/DK/PHS	0.5	1	.375

DK, Direct Kugel; K, Kugel; L, Lichtenstein; MP, mesh‐plug; NS, not significant; NS, not significant; PHS, Prolene Hernia System; PSM, propensity score matched analysis; RCT, randomized controlled trial.

The main limitation of this study was its retrospective nature, which suggests that significant selection bias might exist in the two groups. In this study, open mesh repairs were performed using one of five technical variations; therefore, there was no fully unified procedure. Nevertheless, we used PSM in an attempt to minimize the effects of potential confounding factors. The follow‐up period was also insufficient to clarify the long‐term outcomes, although similar follow‐up periods were used in previous studies. On the other hand, there are interesting data on the timing of recurrence after TEP. Based on previous reports,^25^, ^28^, ^29^, ^30^ over 70% of recurrences occur within the first year after surgery. Although longer follow‐up periods may show increased recurrence rates, the trend can be captured even during the initial 2‐year follow‐up period because most recurrences develop early after surgery. We hope to address these issues with a prospective randomized controlled trial, which would help identify the optimal inguinal hernia repair procedure in our region.

In conclusion, this multicenter retrospective study used PSM and revealed low recurrence rates after TEP and open mesh repair for inguinal hernia. Furthermore, TEP had a low incidence of overall morbidity. Thus, TEP could be a standard procedure for a well‐trained surgical team. A prospective study is necessary to clarify the safety and utility of TEP for inguinal hernia.

## DISCLOSURE

Conflict of Interest: The authors declare no conflicts of interests for this article.
